# Performance Improvement of a Natural Language Processing Tool for Extracting Patient Narratives Related to Medical States From Japanese Pharmaceutical Care Records by Increasing the Amount of Training Data: Natural Language Processing Analysis and Validation Study

**DOI:** 10.2196/68863

**Published:** 2025-03-04

**Authors:** Yukiko Ohno, Tohru Aomori, Tomohiro Nishiyama, Riri Kato, Reina Fujiki, Haruki Ishikawa, Keisuke Kiyomiya, Minae Isawa, Mayumi Mochizuki, Eiji Aramaki, Hisakazu Ohtani

**Affiliations:** 1 Graduate School of Pharmaceutical Sciences Keio University Tokyo Japan; 2 Faculty of Pharmacy Takasaki University of Health and Welfare Takasaki Japan; 3 Nara Institute of Science and Technology Ikoma Japan; 4 Faculty of Pharmacy Keio University Tokyo Japan; 5 Department of Pharmacy Keio University Hospital Tokyo Japan; 6 School of Medicine Keio University Tokyo Japan

**Keywords:** natural language processing, NLP, named entity recognition, NER, deep learning, pharmaceutical care record, electronic medical record, EMR, Japanese

## Abstract

**Background:**

Patients’ oral expressions serve as valuable sources of clinical information to improve pharmacotherapy. Natural language processing (NLP) is a useful approach for analyzing unstructured text data, such as patient narratives. However, few studies have focused on using NLP for narratives in the Japanese language.

**Objective:**

We aimed to develop a high-performance NLP system for extracting clinical information from patient narratives by examining the performance progression with a gradual increase in the amount of training data.

**Methods:**

We used subjective texts from the pharmaceutical care records of Keio University Hospital from April 1, 2018, to March 31, 2019, comprising 12,004 records from 6559 cases. After preprocessing, we annotated diseases and symptoms within the texts. We then trained and evaluated a deep learning model (bidirectional encoder representations from transformers combined with a conditional random field [BERT-CRF]) through 10-fold cross-validation. The annotated data were divided into 10 subsets, and the amount of training data was progressively increased over 10 steps. We also analyzed the causes of errors. Finally, we applied the developed system to the analysis of case report texts to evaluate its usability for texts from other sources.

**Results:**

The *F*_1_-score of the system improved from 0.67 to 0.82 as the amount of training data increased from 1200 to 12,004 records. The *F*_1_-score reached 0.78 with 3600 records and was largely similar thereafter. As performance improved, errors from incorrect extractions decreased significantly, which resulted in an increase in precision. For case reports, the *F*_1_-score also increased from 0.34 to 0.41 as the training dataset expanded from 1200 to 12,004 records. Performance was lower for extracting symptoms from case report texts compared with pharmaceutical care records, suggesting that this system is more specialized for analyzing subjective data from pharmaceutical care records.

**Conclusions:**

We successfully developed a high-performance system specialized in analyzing subjective data from pharmaceutical care records by training a large dataset, with near-complete saturation of system performance with about 3600 training records. This system will be useful for monitoring symptoms, offering benefits for both clinical practice and research.

## Introduction

In clinical settings, information, such as changes in a patient’s condition and the occurrence of adverse events, is essential for providing optimal pharmaceutical care. However, physicians commonly underestimate or underreport symptoms expressed by patients themselves [1,2]. For example, a clinical study involving patients with advanced non–small cell lung cancer showed that physicians underreported grade 2 skin toxicity and fatigue and all grades of diarrhea compared with patients, with only a few grade 3 and grade 4 adverse events reported [2]. In contrast, patient self-reports, including patient questionnaires and the PRO-CTCAE (patient-reported outcomes version of the Common Terminology Criteria for Adverse Events) Measurement System, are considered useful and are increasingly used in cross-sectional surveys of adverse events [3-5]. Therefore, the clinical information contained in a patient’s self-report can be used, when appropriately identified, to prevent an underestimation of patient symptoms.

Unlike structured databases, such as the Japanese Adverse Drug Reaction Report Database, text data, such as patient narratives, comprising natural language, are often unstructured and ambiguous in meaning. To enable computers to handle and analyze natural languages, appropriate natural language processing (NLP) technology is necessary. For example, named entity recognition (NER) technology can be used to extract disease and symptom names from natural language text and determine whether the extracted terms are positively or negatively expressed (positive-negative classification). NER is expected to be useful for analyzing symptoms, as in adverse event monitoring. NER has been applied to data from social media platforms to investigate patient outcomes related to post–COVID-19 conditions [6] and to assess the frequency of adverse drug reactions [7].

Patient narratives are accumulated in pharmacist records as well as posts on social media platforms. Because pharmacists routinely assess the efficacy and safety of pharmacotherapy, their records contain a large amount of patient information regarding adverse drug events. Pharmacist records are often structured using the SOAP format: subjective data (S), objective data (O), assessment (A), and plan (P). The subjective data in the SOAP format contain the patients’ raw words. Therefore, the subjective data in pharmacist records could serve as a rich source of patient narratives related to adverse events.

As mentioned above, some investigations have been conducted to extract information, such as diseases and symptoms, from text data. For example, Nikfarjam et al [7] developed a neural network–based system to extract adverse drug events from patient postings on social health networks in English. Similarly, Batbaatar et al [8] employed deep learning methods to extract not only diseases and symptoms but also pharmacological substances and other health-related information from social media service posts in English. However, most NER research has focused on physician records [9-11] or data in English [6-8,12-18], with few studies targeting Japanese pharmacist records.

In a study focusing on Japanese pharmacist records, Usui et al [19] developed a rule-based system to extract patient complaints from the electronic medication records of Japanese community pharmacies and standardize them using International Statistical Classification of Diseases and Related Health Problems 10th Revision (ICD-10) codes. However, the rule-based system could not handle patient words not appearing in its predefined list of rules. Therefore, we hypothesized that a deep learning model could extract diseases and symptoms from the text more accurately than the rule-based system of Usui et al [19].

A system has already been developed to target physician records based on a deep learning model, bidirectional encoder representations from transformers (BERT)–conditional random field (CRF). BERT is an effective NLP model that can be fine-tuned for a variety of tasks [20]. In addition, several studies have reported that BERT-CRF [21,22], which adds a CRF layer to the output, performs well in the NER task. We previously examined whether the current system could be adapted to pharmaceutical care records without modification [23]. The system showed relatively high performance for the assessment data from pharmaceutical care records written in SOAP format. However, the performance of the system was inadequate for subjective data, objective data, and the plan. Furthermore, taking into account the importance of subjective data in adverse event analysis, it was determined that the system needed to be trained using subjective data.

In this study, we aimed to develop a new NLP system to accurately extract disease and symptom names (disease name extraction) from patients’ subjective data and determine whether these conditions are affirmed or denied (ie, present or absent). In addition, it is generally accepted that system performance improves with the amount of training data used. However, case reports and physician summaries, in which more research has been conducted than patient narratives, are organized in physicians’ own words. On the other hand, patients’ narratives are freely worded and nonorganized. Therefore, it is important to know how the system’s performance changes as the training amount increases when targeting such unorganized data. Furthermore, from an annotation cost perspective, annotation of medical documents requires more cost and effort due to its highly specialized and complicated nature. Therefore, resources can be distributed efficiently by predicting the approximate amount of annotation with a high learning effect. Thus, we also investigated how the system performance changed as the amount of training data increased.

## Methods

### Overview

**Figure 1:**
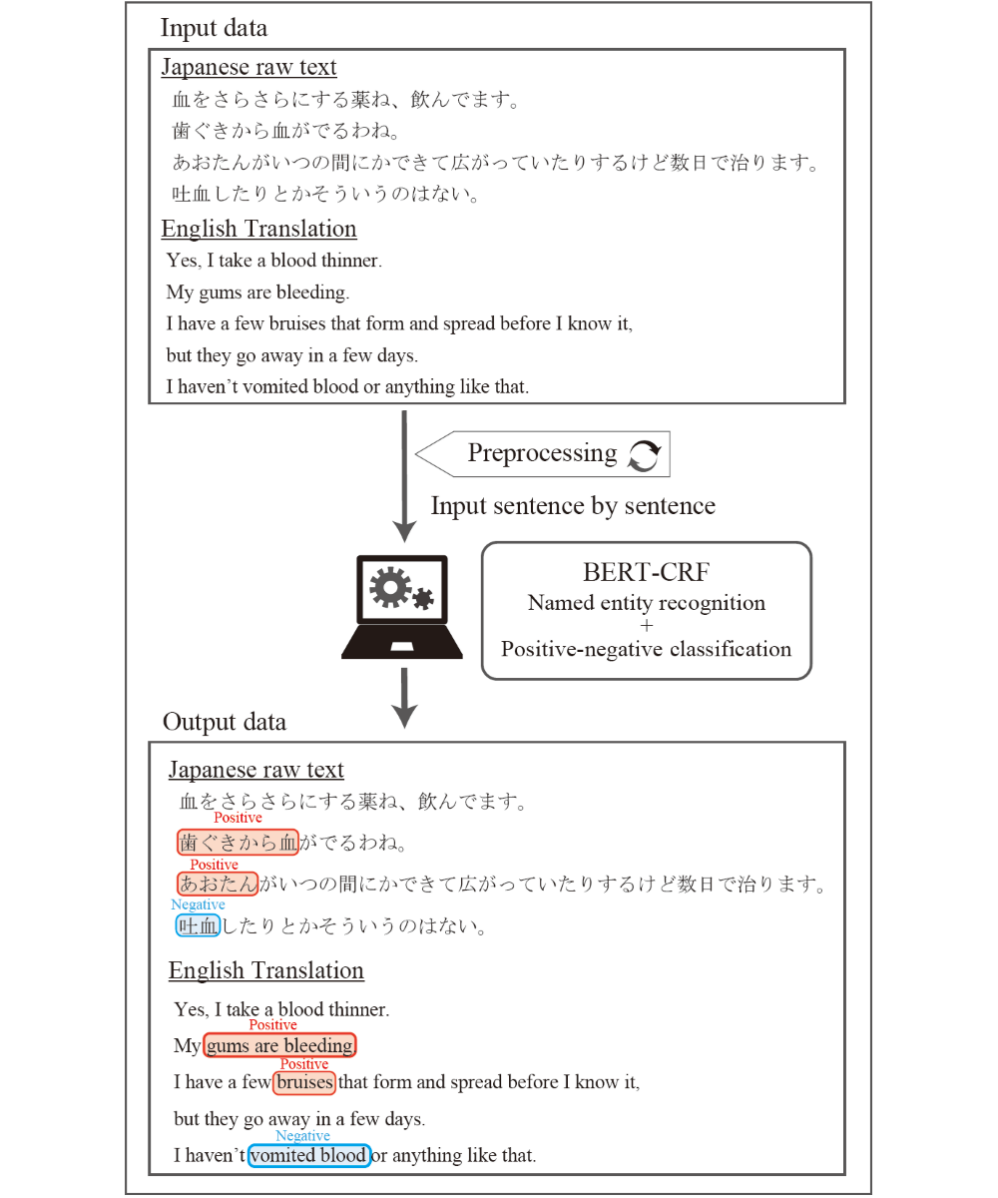
Conceptual diagram of the disease-name extraction system operation. After inputting preprocessed text data into the system sentence by sentence, the system extracts diseases and symptoms as outputs and predicts the presence or absence of each finding. BERT: bidirectional encoder representations from transformers; CRF: conditional random field.

**Figure 2:**
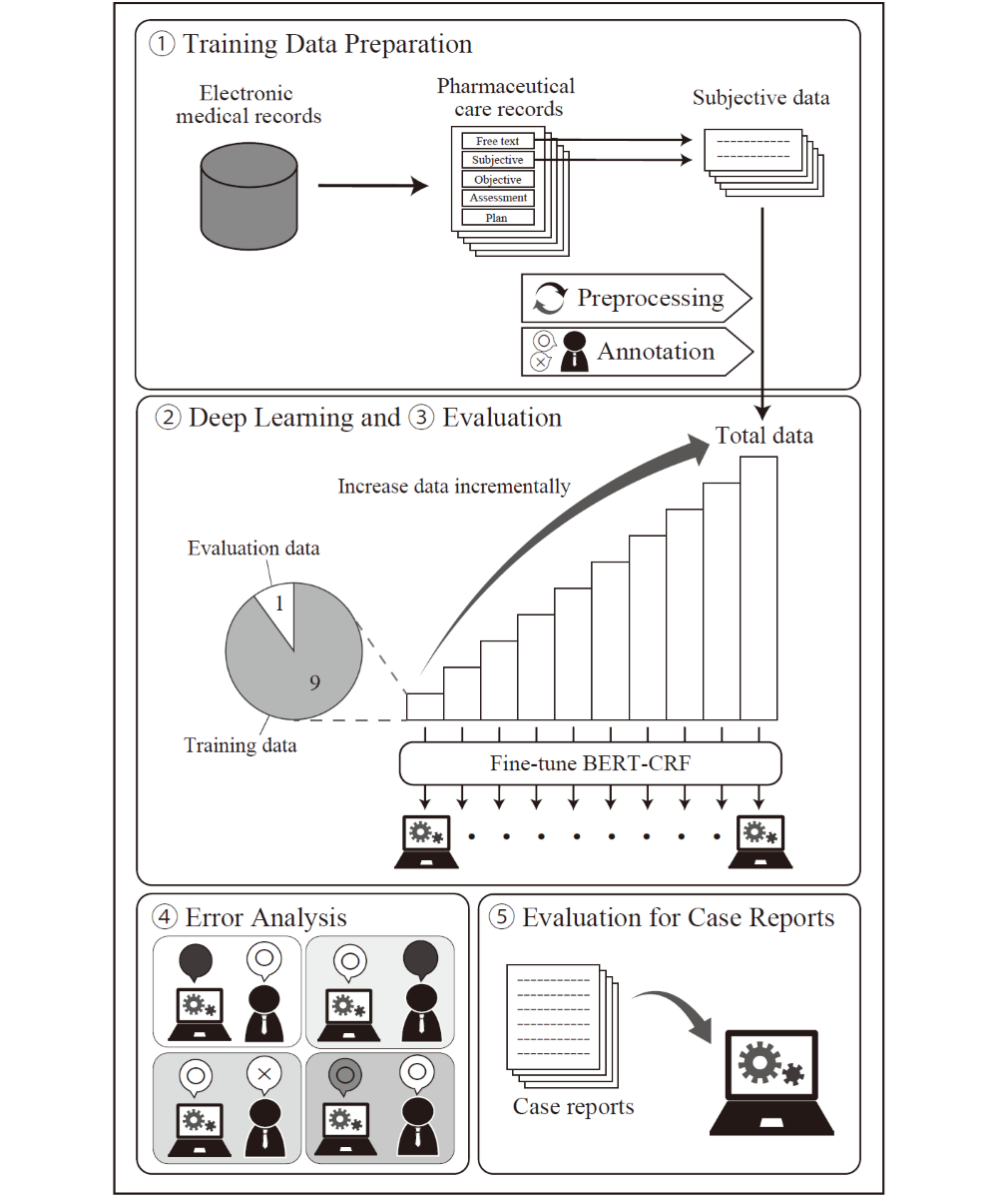
Overview of this study. Subjective data were collected from pharmaceutical care records in the electronic medical record system. Data were preprocessed, and diseases and symptoms in the data were annotated to prepare the training data. A disease-name extraction system was developed, and its performance was evaluated through 10-fold cross-validation with progressive increases in the amount of training data over 10 steps. Errors that occurred during the performance evaluation were sampled, and the causes of the errors were analyzed. The developed system was applied to case reports, and its performance for case reports was investigated. BERT: bidirectional encoder representations from transformers; CRF: conditional random field.

We aimed to develop a system that extracts disease names from patients’ subjective texts and determines whether the conditions are affirmed or denied in the sentence (Figure 1). The input was processed sentence by sentence. To develop and evaluate the system, this study included 5 parts: training data preparation, model training, evaluation, error analysis, and evaluation of the usability of the system for case reports (Figure 2).

In the training data preparation, subjective texts were preprocessed and annotated. In the deep learning part, a disease-name extraction system was constructed based on a deep learning model by increasing the amount of training data in 10 steps. In the evaluation part, cross-validation was used to evaluate performance at the same time as training. We sampled the evaluation results and classified the causes of errors in the error analysis part. In the evaluation of the usability of the system for case reports, we evaluated the performance of the developed system when applied to case reports. Each step has been described in the following sections.

### Training Data Preparation

The pharmaceutical care records in the electronic medical record system of Keio University Hospital, written by pharmacists in Japanese, were used as the source of training data. These records comprise 5 columns: subjective, objective, assessment, plan, and free text. In some cases, SOAP-formatted sentences are included in the free-text column. In this study, we collected 12,004 subjective data records from 6559 cases in the subjective and free-text columns described from April 1, 2018, to March 31, 2019.

All text data in the pharmaceutical care records were converted to full-width (2-byte) characters. Line breaks or periods were used as sentence separators, and spaces at the beginning and end of sentences, as well as blank lines, were deleted. Additionally, the string “_X000D_,” indicating a line break, was removed. Two researchers independently annotated 400 records based on the annotation criteria described in the following paragraph. A comparison between their annotations obtained a high κ coefficient (κ coefficient=0.92), indicating almost perfect agreement. Therefore, the remaining data were annotated by a single researcher.

The annotation criteria were originally developed by our group to collect information for adverse event monitoring in clinical practice and epidemiological studies. Patient expressions related to diseases or symptoms, including nouns, verbs, adjectives, and adverbs, were extracted. The time of onset, site, severity, and triggers of symptoms were also extracted when they were described near the clinical findings. Numerical information was excluded because such data should be obtained from the structured electronic medical record database rather than text data. However, if the text verbally referred to changes in laboratory values, such as “increase in blood pressure,” it was extracted as a target. Texts referring to normal renal function, liver function, blood electrolyte levels, appetite, sleep, and bowel movements were interpreted as negative references to abnormalities (ie, denial of symptoms) and were included in the extraction.

### Deep Learning Method

In this study, we used BERT-CRF as a deep learning method. Several pretrained Japanese BERT models have been developed and published. In this study, we used the character-based BERT of Tohoku University, which is the most commonly used pretrained BERT model for Japanese. BERTJapaneseTokenizer [24], which uses MeCab as a morphological analyzer, was used as a tokenizer. Fine-tuning was performed with a batch size of 32 and with 10-fold cross-validation. In the 10-fold cross-validation, data were shuffled by sentence and then split into 10 groups (folds). The amount of training data was increased in 10 steps from 1200 to 12,004 records.

### Evaluation

Precision, recall, and *F*_1_-score were used as indices for performance evaluation. Precision was calculated as follows: number of true positives/number of true positives and false positives. Recall was calculated as follows: number of true positives/number of true positives and false negatives. *F*_1_-score was calculated as follows: 2 × precision × recall/(precision + recall).

The final accuracy was calculated with the cross-validation method. The number of epochs was determined based on the *F*_1_-scores obtained during cross-validation. We also investigated the performance by taking partial matches into consideration, as partially matched terms can still convey valuable clinical information. The Levenshtein distance [25] was used as the criterion for partial matches. The Levenshtein distance measures the resemblance between 2 strings (W1 and W2) and is defined as the minimum number of character deletion, insertion, and replacement operations required to convert one string into the other. Similarity was calculated using the following formula: similarity (W1, W2) = {max (|W1|, |W2|) − Levenshtein distance}/max (|W1|, |W2|), where |Wn| represents the number of characters (length) of Wn. Similarity values ranged from 0 to 1. We sampled the results of the cross-validation to list the partial matches and investigated any failures to extract essential terms that were correctly detected by researchers due to partial matches. Detailed results of this survey are provided in Multimedia Appendix 1. In this survey, the number of incomplete extractions without essential terms increased rapidly when the similarity was below 0.667. Of the 63 extracted terms with a similarity of 0.667 or higher, 4 terms had partially or entirely missing essential terms. However, at the next lowest similarity levels of 0.636 and 0.625, 5 of 9 extracted terms had partially or entirely missing essential terms. Therefore, in this study, “the number of matches, including partial matches” was defined as the sum of the number of complete matches and partial matches with a similarity of 0.66 or greater. In the error analysis, “partial matches with a lower similarity,” described in the next section, were defined as partial matches with a similarity of less than 0.66. During the process of information processing in BERT, infrequent characters were converted to unknown keys ([UNK]). Because the data that we used to evaluate partial matches contained [UNK], we converted all [UNK] into full-width asterisks to calculate the similarity of the Levenshtein distances by regarding them as single characters.

### Error Analysis

For 5 steps of the amount of training data, the results of the system were compared with the annotated diseases and symptoms in the validation data, and the causes of mismatches were classified into 4 categories (Table 1): error 1, failure of the system to extract; error 2, incorrect extraction by the system; error 3, difference in positive-negative classification; and error 4, partial matches with low similarity. Because the amount of data contained in 1 fold was different for each training amount, we used the number of folds that contained close to 1200 records: 10 folds, 3 folds, 2 folds, 1 fold, and 1 fold in the training data of 1200, 3600, 6002, 8402, and 12,004 records, respectively. The choice of which fold of the validation data to use for this analysis was based on the proximity of the *F*_1_-score to the average rates when they were used in the evaluation. In this study, we defined the total number of extractions by the following formula: total number of extractions = number of researchers’ extractions + number of system extractions − (number of exact matches + number of partial matches with a similarity of 0.66 or greater). The error rate was given as the ratio of the number of errors to the total number of extractions: error rate (%) = number of errors × 100/total number of extractions.

**Table 1:** Definitions of error cause categories.

P-N^a^ classification	Named entity recognition			
	Exact match	Partial match		Unmatch
		1>similarity>0.66	0.66>similarity>0	
Match	Exact match	Partial match	Error 4^b^	Error 1^c^/2^d^
Unmatch	Error 3^e^	Error 3^e^	Error 1^c^/2^d^	Error 1^c^/2^d^

^a^P-N: positive-negative.

^b^Error 4: partial matches with low similarity.

^c^Error 1: system extraction failure.

^d^Error 2: incorrect extraction by the system.

^e^Error 3: difference in the P-N classification.

In cases where the number of extracted terms differed between the researchers and the system, the number of errors was counted for the larger number. For example, when one extracted 2 symptoms and the other extracted them as a single (long-named) symptom, we counted them as 2 errors. Therefore, the sum of the error rate and the correct answer rate could exceed 100%. We further investigated changes in the error rates of the 4 categories with an increase in the amount of training data. For error categories with a greater increase in variation of the error rate obtained by the following formula, a detailed descriptive error analysis was performed: variation of error rates (%) = error rates after increasing training amount/error rates before increasing training amount × 100.

When the error rate before increasing the training amount was 0% and the error rate after increasing the training amount was greater than 0%, the variation of error rates was regarded as 100%.

### Evaluation for Case Reports

To evaluate whether the developed system can be applied to other types of texts, we assessed the system performance using Japanese case report data. A series of 148 case reports was collected as validation data from the Real-MedNLP Test Collection. These data were distributed in the Real-MedNLP Test Collection and permitted for redistribution by the respective journal publisher [10]. These reports are available as open access through the “Japan Science and Technology Information Aggregator, Electronic,” an electronic journal platform operated by the Japan Science and Technology Agency.

Case report validation data were also preprocessed and annotated in the same manner as the training data. Two researchers independently annotated 15 cases, approximately 10% of the total cases. Because the agreement between their annotations was almost perfect, with a κ coefficient of 0.93, the remainder was annotated by a single researcher. The validation data were applied to the 10 systems that were developed in the 10-fold cross-validation of each training amount to evaluate performance. The individual performances of the 10 systems were averaged and regarded as the performance of the system for each training amount.

### Ethical Considerations

This study was conducted with the approval of the Keio University School of Medicine Ethics Committee (approval number: 20200067).

An opt-out method was used to ensure that study subjects had the opportunity to refuse the use of their information, since obtaining informed consent was difficult due to the large number of subjects and the fact that many of them had already been discharged from the hospital and had no direct contact with the hospital. Information on the research has been disclosed on the website of the Clinical Translational Research Center, Keio University Hospital.

Each subject’s electronic medical record information was assigned a dummy ID at the time of registration, and information was managed through pseudonymization using dummy IDs.

## Results

### Training Data Features

12,004 subjective data in the pharmaceutical care records used as training data contained 43,553 sentences. These records included 12,287 affirmed diseases and symptoms and 8822 denied diseases and symptoms.

### Evaluation

**Figure 3:**
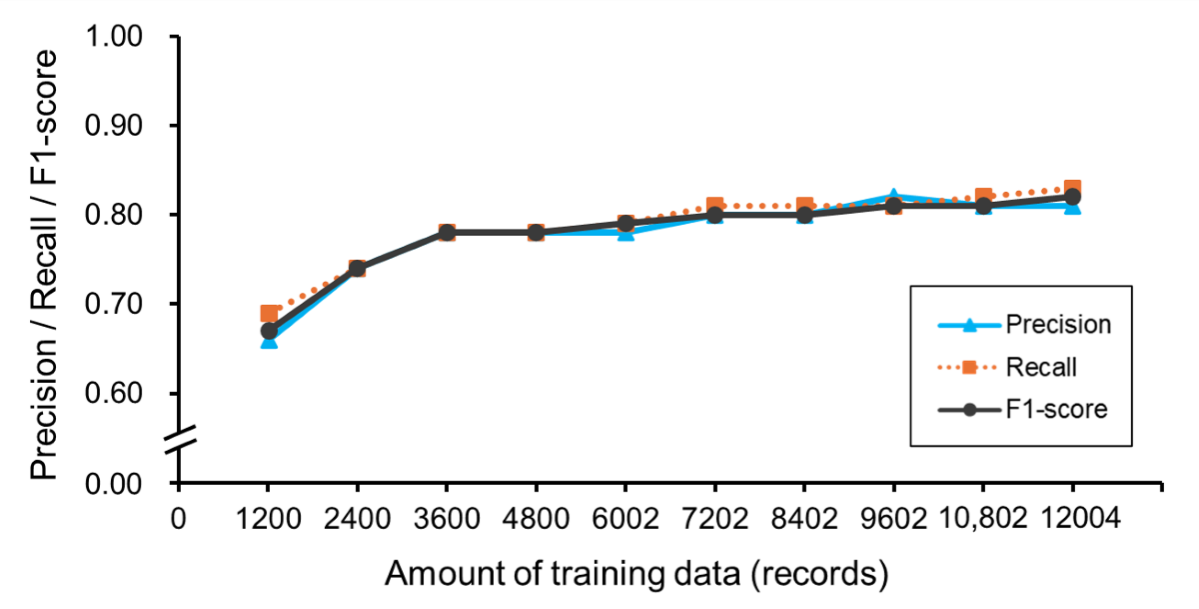
Training curve during cross-validation (only exact matches). Trends in the mean precision, recall, and F1-score values are shown from 1200- to 12,004-record trainings.

**Figure 4:**
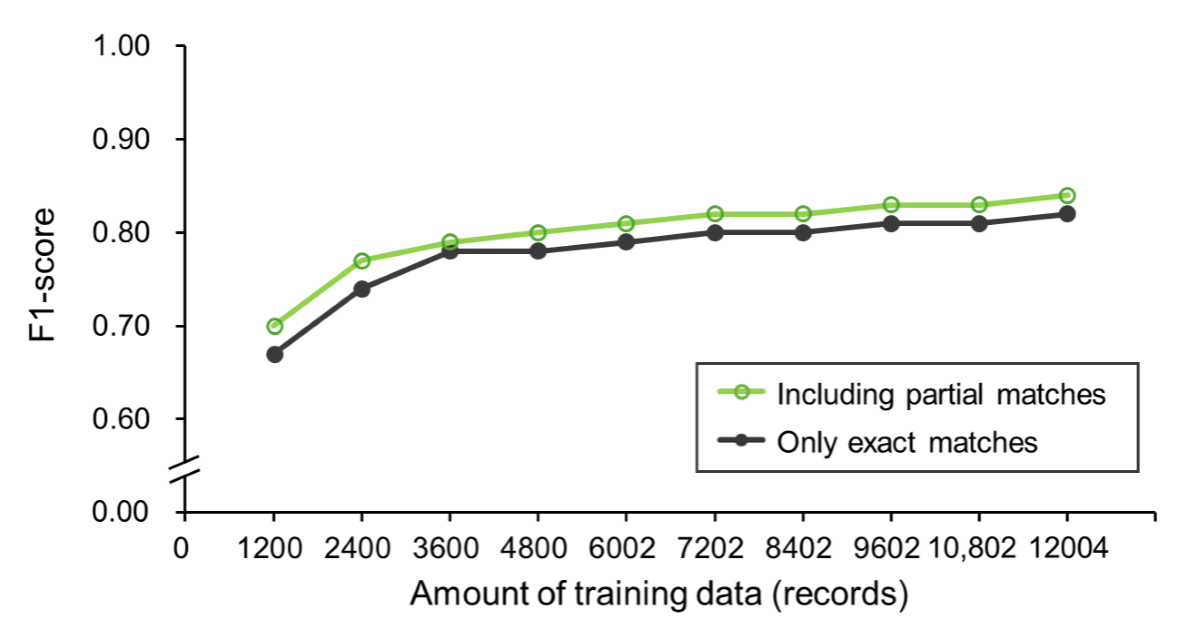
Training curve of the F1-scores during cross-validation (only exact matches/including partial matches). Trends in the mean F1-scores in which only exact matches were considered matches and in the mean F1-scores in which partial matches were included as matches are shown from 1200- to 12,004-record trainings.

The training curves for precision, recall, and *F*_1_-score values for exact matches are shown in Figure 3. As the training data increased from 1200 to 12,004 records, precision, recall, and *F*_1_-score improved from 0.66 to 0.81, from 0.69 to 0.83, and from 0.67 to 0.82, respectively. A power approximation expression for the *F*_1_-score of exact matches yielded the equation: y = 0.40x^0.08^, predicting an *F*_1_-score of 0.90 with 25,251 training records. Performance improvement saturated after training with 3600 records, indicating that a large amount of training data is needed to improve the *F*_1_-score beyond 0.78. Comparing the *F*_1_-scores for exact matches and those including partial matches, the performance gap tended to diminish as the amount of training data increased (Figure 4). The system trained with 12,004 records achieved an *F*_1_-score of 0.82 for exact matches and 0.84 when partial matches were included.

### Error Analysis

**Figure 5:**
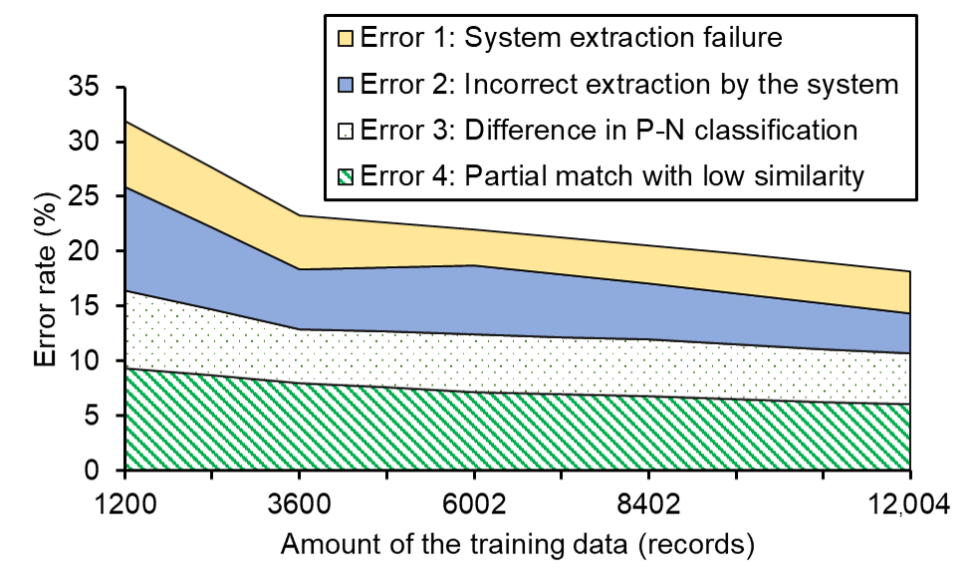
Trends in error rates for each error cause category. A sample study of performance evaluation results was conducted from 1200- to 12,004-record trainings. The errors are classified into 4 cause categories, and trends in error rates for each category are shown. P-N: positive-negative.

Error analysis was carried out for 4395, 3924, 4396, 3071, and 4356 sentences from the validation data during cross-validation using 1200, 3600, 6002, 8402, and 12,004 records, respectively. The causes of errors were classified, and Figure 5 shows the transition of error rates for each cause. The overall error rate decreased from 31.9% (945/2963) to 18.2% (444/2446). The rate of error 2 (incorrect extraction by the system) showed the most significant decrease with an increase in the training records, followed by errors 4, 3, and 1. Error 2 decreased from 9.4% (280/2963) to 3.7% (90/2446) (–5.7 percentage points). Errors 4, 3, and 1 decreased from 9.4% (278/2963) to 6.1% (148/2446) (–3.3 percentage points), from 7.1% (209/2963) to 4.7% (114/2446) (–2.4 percentage points), and from 6.0% (178/2963) to 3.8% (92/2446) (–2.2 percentage points), respectively. Error 4 was the primary cause of error for the system trained with 12,004 records, followed by errors 3, 1, and 2. The 3600-record training when performance reached a plateau was used as a reference point. Comparing the variation in the error rates associated with the increase in the training amount from 1200 to 3600 records to the variation in the error rates associated with the increase in the training amount from 3600 to 12,004 records, the variation for errors 1 and 4 decreased by 9 percentage points and 5 percentage points, respectively, while the variation for errors 2 and 3 increased by 24 percentage points and 10 percentage points, respectively, indicating that improvement was stagnant. Therefore, we categorized errors 2 and 3 into subcategories and investigated the difference in the variation in error rates before and after the 3600-record training.

Error 2 was further classified into 7 subgroups (Table 2). The most common errors in the 1200-record training involved error 2a (the extraction of information that was neither symptoms nor supplemental information related to symptoms). This subcategory encompassed a wide variety of patient expressions, including behaviors, such as “strained in the bathroom,” indications of healthiness, such as “my blood pressure has been quite good,” and medication-related issues unrelated to patient symptoms, such as “the remaining number (of medications) became limited.” The second most common subcategory, error 2b (incomplete extraction without essential terms), involved errors in extracting terms that were not considered symptoms due to the absence of a subject, such as “loose” or “didn’t go well with me.” It also included cases where only supplemental words were extracted, such as “at night” or “very much.” The third most common subcategory, error 2c, involved errors related to the extraction of conditional terms that were annotated or not annotated by researchers depending on the context or were not finally annotated after the researcher was faced with a difficult decision. For example, researchers would annotate the patient expression “I couldn’t eat” as it indicates a symptom of the loss of appetite, while the expression “I couldn’t eat because I was too sleepy” would not be attributed to appetite. In addition, the expression “my stomach went like this” may have been regarded as a symptom because it represented an abnormal condition. However, the researchers finally decided not to annotate it. The next most common error subcategories were errors 2d and 2e (partial matches with low similarity and an incorrect positive-negative classification), which involved either entire or partial extraction of essential terms.

**Table 2:** Number of errors and the ratio of errors to total extraction in the subcategories of error 2.

Subgroup	Number of errors in 1200-record training (n=2963), n (%)	Number of errors in 3600-record training (n=2387), n (%)	Number of errors in 12,004-record training (n=2446), n (%)
Total errors of category 2	280 (9.4)	130 (5.4)	90 (3.7)
Error 2a: Extraction of information that was neither symptoms nor supplemental information	86 (2.9)	32 (1.3)	12 (0.5)
Error 2b: Incomplete extraction without essential terms	76 (2.6)	27 (1.1)	16 (0.7)
Error 2c: Extraction of conditional terms that were annotated or not by researchers depending on the context or were not finally annotated after the researcher was faced with a difficult decision	55 (1.9)	47 (2.0)	32 (1.3)
Error 2d: Partial matches with low similarity and incorrect P-N^a^ classifications that involved entire extractions of essential terms	35 (1.2)	15 (0.6)	12 (0.5)
Error 2e: Partial matches with low similarity and incorrect P-N classifications that involved partial extractions of essential terms or incomplete extractions without essential terms	19 (0.6)	2 (0.08)	3 (0.1)
Error 2f: Symptoms that the researchers forgot to extract	7 (0.2)	6 (0.3)	12 (0.5)
Error 2g: Partial matches with low similarity involving entire extractions of symptoms for which the researchers made incorrect P-N classifications	2 (0.1)	1 (0.04)	3 (0.1)

^a^P-N: positive-negative.

From 1200- to 12,004-record trainings, significant improvements were observed in the following order: “extraction of information that was neither symptoms nor supplemental information (error 2a),” “incomplete extraction without essential terms (error 2b),” and “partial matches with low similarity and incorrect positive-negative classifications that involved entire extractions of essential terms (error 2d).” In contrast, “symptoms that the researchers forgot to extract (error 2f)” and “partial matches with low similarity involving entire extractions of symptoms for which the researchers made incorrect positive-negative classifications (error 2g)” increased from 0.2% (7/2963) to 0.5% (12/2446) and from 0.1% (2/2963) to 0.1% (3/2446), respectively. However, other subgroups showed overall improvements.

The differences in the variation of each subcategory before and after the 3600 cases were large in the following order: “partial matches with low similarity involving entire extractions of symptoms for which the researchers made incorrect positive-negative classifications (error 2g)” (231 percentage points), “partial matches with low similarity and incorrect positive-negative classifications that involved partial extractions of essential terms or incomplete extractions without essential terms (error 2e)” (133 percentage points), “symptoms that the researchers forgot to extract (error 2f)” (89 percentage points), “partial matches with low similarity and incorrect positive-negative classifications that involved entire extractions of essential terms (error 2d)” (25 percentage points), “incomplete extraction without essential terms (error 2b)” (14 percentage points), “extraction of information that was neither symptoms nor supplemental information (error 2a)” (–10 percentage points), and “extraction of conditional terms that were annotated or not by researchers depending on the context or were not finally annotated after the researcher was faced with a difficult decision (error 2c)” (–40 percentage points). A larger difference in the variation indicates a relative stagnation in the improvement of errors on increasing the training amount beyond 3600 records.

Error 3 was classified into 5 subcategories (Multimedia Appendix 2). The top 3 subcategories with the largest differences in variation before and after the 3600-record training in each subcategory were as follows: “determined from positive expressions (error 3b)” (125 percentage points), “determined from negative expressions (error 3c)” (64 percentage points), and “determined from other information (error 3a)” (–8.4 percentage points).

The contents of errors 3b and 3c, which showed particularly large differences in variation rates, were classified into further subcategories based on 2 points: the position of the positive-negative expression and the way in which the positive-negative expression was stated (Multimedia Appendix 3 and 4). Based on the method focusing on the position and the way of positive-negative expressions, we classified them into 5 and 6 subcategories, respectively. The differences in the variation before and after the 3600-record training in the subcategories focusing on the position of positive-negative expressions were large in this order: “more than three words are in between from the extracted terms (error 3bc-1-4)” (369 percentage points), “determination with reference to the positive-negative classification of another extracted term in the same sentence (error 3bc-1-5)” (200 percentage points), “included within the extracted terms (error 3bc-1-2)” (195 percentage points), “one or two words are in between from the extracted terms (error 3bc-1-3)” (90 percentage points), and “immediately before or/and after the symptom (error 3bc-1-1)” (60 percentage points).

The differences in the variation before and after the 3600-record training in the subcategories focusing on the way of positive-negative expressions were large in this order: “determination with reference to the positive-negative classification of another extracted term in the same sentence (error 3bc-2-5)” (200 percentage points), “reversal of positive-negative classification by partial match (error 3bc-2-6)” (200 percentage points), “included within the extracted terms (error 3bc-2-2)” (157 percentage points), “mild negative expressions (error 3bc-2-3)” (119 percentage points), “simple positive or negative expressions (error 3bc-2-1)” (76 percentage points), and “negated positive or negative expressions (error 3bc-2-4)” (–15 percentage points).

“Error 3bc-1-5 (3bc-2-5): determination with reference to the positive-negative classification of another extracted term in the same sentence” and “error 3bc-2-6: reversal of positive-negative classification by partial match” had an error rate of 0% at the 3600-record training. Therefore, the difference in variability was large, as only one or two errors at the 12,004-record training resulted in 100% variation.

### Evaluation of Case Reports

**Figure 6:**
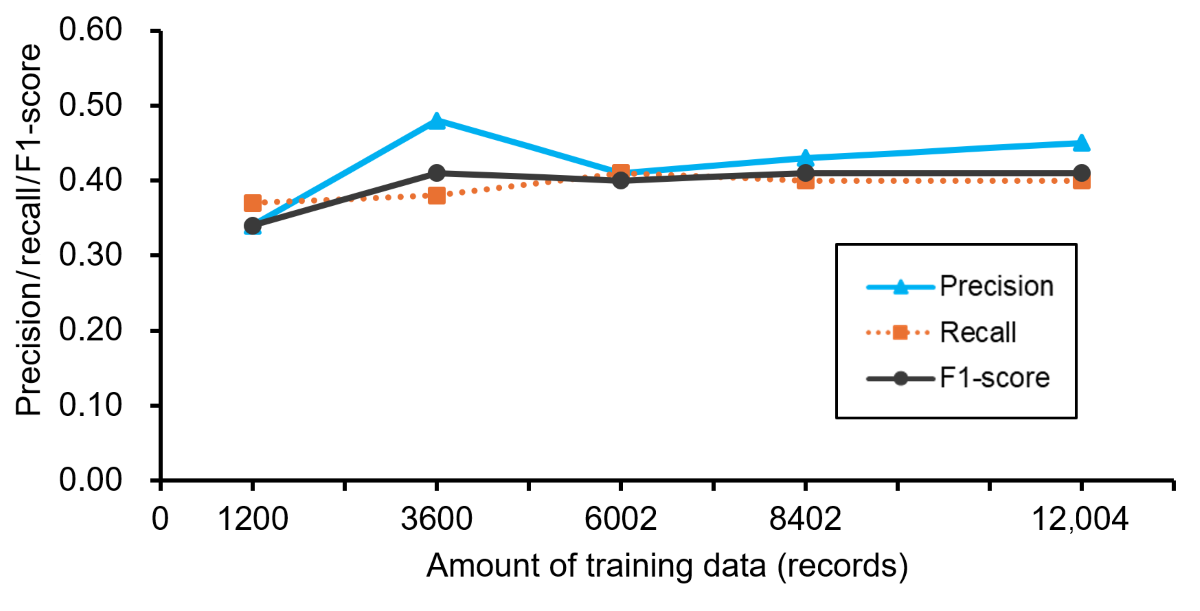
Performance of the case report analysis (only exact matches). Case reports were analyzed using systems trained with between 1200 and 12,004 pharmaceutical care records. Trends in the mean precision, recall, and F1-score values, which included only exact matches as matches, are shown.

**Figure 7:**
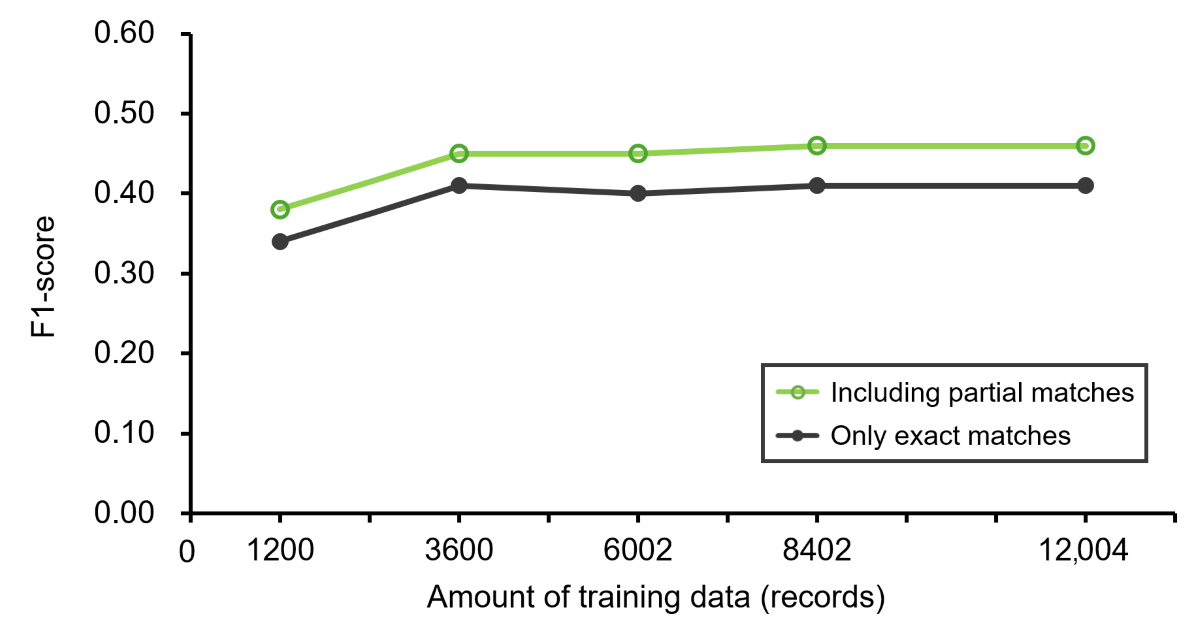
F1-scores of the case report analysis (only exact matches/including partial matches). Case reports were analyzed using systems trained with between 1200 and 12,004 pharmaceutical care records. Trends in the mean F1-scores in which only exact matches were considered matches and in the mean F1-scores in which partial matches were included as matches are shown.

Figure 6 shows the improvement in system performance for the handling of the text data of case reports with an increase in the number of training datasets. The *F*_1_-score increased from 0.34 to 0.41 with an increase in the training dataset and saturated at 3600 records of the training dataset. The differences between the *F*_1_-scores for case reports and those for pharmaceutical care records were 0.33 and 0.41 at 1200 and 12,004 training dataset records, respectively. The differences between the *F*_1_-scores for exact matches and those including partial matches were larger than those at cross-validation. In the performance evaluation for case reports, the *F*_1_-scores for partial matches were always 0.04-0.05 higher than those for exact matches (Figure 7).

## Discussion

### Principal Findings

We developed a disease-name extraction system targeting subjective data in Japanese pharmaceutical care records by fine-tuning BERT-CRF. The performance of the system, evaluated by exact matches with a correct positive-negative classification, improved as the amount of training data increased, even though the system targeted subjective data, which has not been adequately addressed before. A similar trend was observed when evaluated by matches including partial matches, with a similarity score of 0.66 or higher. Although there was a certain number of partial match extractions, the extraction performance with partial matches was close to that for exact matches. This could be attributed to the strict criterion for similarity used in this study. The system performance increased slowly and reached a plateau at the 3600-record training. The results were presumably influenced by a combination of factors, including the deep learning model used (BERT-CRF), the complex annotation criteria, and human error in the annotation, which cannot be determined from this study. To resolve this point and further improve system performance, the use of deep learning models other than BERT-CRF should be considered. In addition, system performance will vary depending on the content of the training data. Therefore, when other training data are used to develop the system, it is difficult to say whether the system performance will reach a plateau with the same training amount in this study. However, by utilizing the characteristics of a gradual curve in performance transition, efficient training can be achieved by increasing the amount of training data step by step from a small amount and using an approximate formula to predict the training amount when performance reaches a plateau.

The analytical performance of the system was lower for case reports than for pharmaceutical care records. When records from different sources were used as training data, the improvement in system performance with increasing training data was inferior to that observed when training with records from the same source type. In addition, the analytical performance gap between text from the same type of training data and that from different types of data persisted, regardless of the amount of training data. In other words, the developed system was not well-suited for analyzing case reports but proved especially useful for analyzing patient narratives when trained on subjective data from pharmaceutical care records. This indicates that separate system development is necessary to analyze different types of records. The difference in performance between the case report analysis and the pharmaceutical care record analysis is assumed to be largely due to the existence of raw patient statements. However, another important feature is the different occupations of the health care professionals who create the records. Physicians create case reports covering the patients’ conditions comprehensively and summarizing the patients’ backgrounds and disease processes in their own words, while pharmacists mainly monitor drug efficacy and side effects and create pharmaceutical care records. Therefore, pharmacists naturally create SOAP format records that contain a lot of information about adverse events. We would like to emphasize again that our system is likely to be suitable for the collection of adverse event information since the system learned from the subjective data in pharmaceutical care records. Furthermore, since the pharmaceutical care records were written in SOAP format, we were able to selectively collect subjective data and use them for the training data in this study. As a result, a system specialized for subjective data was efficiently developed. This suggests that SOAP-format data may be a useful source for deep learning.

Error analysis revealed that the reduction in error 2 with increased training data was 5.7 percentage points (from 9.4% to 3.7%), a larger decrease than that observed for any other error type. This reduction in false-positive extractions significantly contributed to the improvement in precision. Although we prioritized recall over precision during system development to avoid missing symptoms, the increase in training data led to higher precision without compromising recall. Therefore, we believe that increasing the training data helped to develop a more user-friendly system with fewer false extractions.

### Error Analysis

While errors in most subgroups of error 2 decreased, there were increases in error 2f (symptoms that the researchers forgot to extract) and error 2g (partial matches with low similarity involving entire extractions of symptoms for which the researchers made incorrect positive-negative classifications). Researchers may be more prone to making annotation mistakes as the amount of data to be prepared increases and as they encounter a wider variety of expressions. However, the percentage of these errors in total extractions was only 0.3% (9/2963) for the 1200-record training and 0.6% (15/2446) for the 12,004-record training, and thus, their impacts on overall system performance were small. Additionally, the system showed improvements in other types of false positives, indicating that increasing the training data can help to reduce a wide variety of false-positive errors.

Next, we looked at the variation in error rates for error 2. Among errors 2g, 2e, 2f, and 2d, which showed large differences in error rate variation, “partial matches with low similarity involving entire extractions of symptoms for which the researchers made incorrect positive-negative classifications (error 2g)” and “partial matches with low similarity and incorrect positive-negative classifications that involved partial extractions of essential terms or incomplete extractions without essential terms (error 2e)” were almost eliminated at the 3600-record training. Errors related to “symptoms that the researchers forgot to extract (error 2f)” and “partial matches with low similarity and incorrect positive-negative classifications that involved entire extractions of essential terms (error 2d)” were caused by researchers’ mistakes or errors in the extraction range or positive-negative classification, but the system was able to partially recognize the information to be extracted. Therefore, these errors may have been difficult to resolve.

Among errors 2b, 2a, and 2c, which showed limited differences in error rate variation, the extracted terms or pre/post descriptions of “extraction of information that was neither symptoms nor supplemental information (error 2a)” and “incomplete extraction without essential terms (error 2b)” were different from the real extraction target. Concerning the system’s context-readable specification, the system appears to have learned that the contexts before and after the incorrect extractions were not symptom-related contexts, reducing errors 2a and 2b, as it learned from large amounts of training data. “Error 2c” error rates increased at the 3600-record training. This finding indicates that error 2c improved less than error 2a and 2b when comparing the 1200 and 12,004-record trainings, although the difference in variation was small. Error 2c is more difficult to improve than errors 2a and 2b, presumably because error 2c contains expressions that researchers are also unsure about and that may be extracted depending on the situation.

Among the subcategories of error 3 focusing on the position of positive-negative expressions, “included within the extracted terms (error 3bc-1-2)” (195 percentage points) had a larger difference in error rate variation than “one or two words are in between from the extracted terms (error 3bc-1-3)” (90 percentage points) and “immediately before or/and after the symptom (error 3bc-1-1)” (60 percentage points). This suggests that the system may be able to perform correct positive-negative classification when the extracted terms and the positive-negative expressions are close to each other but not contained within the extracted terms.

Among the subcategories of error 3 focusing on the way of positive-negative expressions, simple sentences were considered easy to judge: “simple positive or negative expressions (error 3bc-2-1)” (76 percentage points) and “negated positive or negative expressions (error 3bc-2-4)” (–15 percentage points). In “mild negative expressions (error 3bc-2-3)” (119 percentage points), on the other hand, the researchers focused on the description of the degree of denial to determine whether the symptoms were completely denied. However, due to the variety of degree descriptions, this annotation criteria could have been cumbersome for the system.

Through this error analysis, it was inferred that while the system can easily improve the extraction results with reference to the context near the extracted terms due to increased learning, the system cannot handle cases where the researchers are unsure of the decision or complex annotation criteria in which the extraction results change depending on the situation.

### Evaluation

The system developed in this study performed better than the systems in previous studies focused on the Japanese language. In a previous study of Japanese patient narratives, Usui et al [19] aimed to thoroughly extract patient complaints from subjective data using a rule-based system. The high performance achieved by our deep learning–based system suggests that our system had considerable flexibility in handling data where symptoms could not be extracted through rules alone. As a result, the performance of our system, with an *F*_1_-score of 0.82, surpassed that of the system by Usui et al [19] (*F*_1_-score of 0.65), although it should be noted that the output functions of the 2 systems were not exactly the same, complicating a direct comparison. Similarly, Aramaki et al [26] used Japanese case reports as training data to develop a system for extracting a variety of symptoms. The *F*_1_-scores of their system were 0.87 for NER and 0.63 for NER with positive-negative classification [26], both of which were lower than the scores achieved by our system. Although many previous studies of NER have focused on specific symptoms or patient populations, our system has the advantage of covering a broader range of patients and extracting a wider variety of symptoms. This makes it particularly useful for comprehensively collecting information on patient narratives.

The performance of the developed system is the same or even higher than that of similar systems in English, which aimed to extract a variety of symptomatic adverse drug events from patient narratives. For example, the neural network–based system developed by Nikfarjam et al [7] achieved a micro-average *F*_1_-score of 0.74 in NER for drug adverse events in patient posts on social health networks. Another system by Batbaatar et al [8] achieved an *F*_1_-score of 0.82 for NER of diseases or syndromes and an *F*_1_-score of 0.88 for signs and symptoms from social media posts. Of these 2 systems, one extracted only the adverse events experienced by the patient, while the other did not involve positive-negative classification. Therefore, our system, despite its added complexity with positive-negative classification, appears to have comparable or even superior performance compared with the aforementioned systems.

### Future Applications

The developed system is expected to be applied to monitor adverse events based on patient reports and narratives. Among previous studies using NER systems, the study by Ujiie et al [27] aimed to reduce the screening burden of drug safety information for pharmaceutical companies by identifying medical articles that contained descriptions of adverse drug events. Similarly, the system developed by Nishioka et al [28] sought to detect a specific adverse reaction—hand-foot syndrome—by identifying blog posts from patients with breast cancer, analyzing each text and post to determine whether it involved the adverse reaction. However, they did not use an NER approach. Given that our system has the advantage of extracting a wide variety of symptoms from diverse patient expressions, it is better positioned for broader use in monitoring adverse events across a wider range of conditions.

Although we focused on patient narratives, assessments recorded by health care professionals are undoubtedly another important source of patient information. Specifically, patient summaries, such as discharge and transfer summaries, contain information that has been reviewed, selected, and organized by health care professionals. However, evaluations by health care professionals carry the risk of underestimation and underreporting [1,2]. When using any medical records for research, including pharmaceutical care records, there is a concern about the filtering of information by health care professionals. However, we succeeded in establishing a system for analyzing patient narratives. The next step is to target patient narratives automatically transcribed by voice input so that the analysis of patient narratives can be achieved with fewer omissions in the recordings. By comparing patient narratives collected by either method with professional assessments using a system, such as ours, the gap between patients and health professionals can be identified. The developed system could contribute to achieving gap-free assessment and care between patients and health care professionals through the identification and analysis of the gap.

### Limitations of This Study

One limitation of this study is that we used data from a single facility. Therefore, further verification is needed to determine whether this system can be used for subjective data from other facilities. Since subjective data include raw patient speech, differences in dialect and the patient’s disease and treatment are assumed to be factors that cause differences in the content of the descriptions between facilities. Therefore, while this study used data from an acute care hospital in Tokyo, the system may perform differently for data from chronic care hospitals, community pharmacies, and medical facilities in areas where dialects are spoken. Validation is expected to be conducted in the future using data from medical facilities with the same features and different features to reveal the versatility of the system.

Another issue is the evaluation of the validity of the system in clinical settings. For example, *F*_1_-score, the evaluation index used in this study, provides a relative evaluation and can be used to compare systems under the same conditions. Since there is no standard value for judging practicality, the results cannot be used to judge the applicability in actual clinical settings. However, since different applications require different performances, the system is potentially usable in a real clinical setting even if it does not have 100% performance. For example, signal detection is more important to capture overall trends than to overlook individual cases, so the system is useful for processing large amounts of data. Another limitation is the lack of standardization for extracted terms, which could be addressed by developing additional databases or systems.

### Conclusions

This is the first study to develop an NER system to extract disease and symptom names from subjective data in Japanese hospital pharmaceutical care records and to investigate how its performance improves with increasing training data. The developed system demonstrated high performance in extracting disease and symptom names when trained on a large dataset, although performance improved gradually as the training amount increased, reaching a plateau after training on approximately 3600 records.

The system enables the monitoring of various symptoms using patient narratives as a source and is expected to be a valuable tool for supporting both clinical practice and research.

## Appendix

Multimedia Appendix 1 Results of the sample survey for the determination of the Levenshtein distance similarity threshold.

Multimedia Appendix 2 Number of errors and the ratio of errors to total extraction in the subcategories of error 3.

Multimedia Appendix 3 Number of errors and the ratio of errors to total extraction in the subcategories of error 3b and 3c focused on the position of positive-negative expressions.

Multimedia Appendix 4 Number of errors and the ratio of errors to total extraction in the subcategories of error 3b and 3c focused on the way of positive-negative expressions.

## Abbreviations

BERT: bidirectional encoder representations from transformers
CRF: conditional random field
NER: named entity recognition
NLP: natural language processing
UNK: unknown keys
