# Supplementary material for: Performance Improvement of a Natural Language Processing Tool for Extracting Patient Narratives Related to Medical States From Japanese Pharmaceutical Care Records by Increasing the Amount of Training Data: Natural Language Processing Analysis and Validation Study
Source: JMIR Med Inform. 2025 Mar 4;13:e68863. doi: 10.2196/68863 (PMC11920660; doi:10.2196/68863)
Supplement: Multimedia Appendix 1 [file medinform_v13i1e68863_app1.docx]

Multimedia Appendix 1. Results of the sample survey for the determination of the Levenshtein distance similarity threshold.

| Terms extracted by the system | Terms extracted by researchers | Symptom (+) | Similarity |
| --- | --- | --- | --- |
| 足の方が | しびれは足 |  | 0.000 |
| 夜 | 夜はトイレに行きたくて＊時間おきに起き |  | 0.0526 |
| ＊ | 夜はトイレに行きたくて＊時間おきに起き |  | 0.0526 |
| 熱が出やすい | アレディアを投与するときは熱 | + | 0.0714 |
| 抗 | 抗がん剤始まってから歯が浮く |  | 0.0714 |
| 胃があれやすい | ロキソニンを飲むと胃があれ | + | 0.0769 |
| 下 | 血圧は入院してから下がって |  | 0.0769 |
| 顔のあたりのむくみがなかなか引かなく | ステロイドこの前使ったら顔のあたりのむくみ | + | 0.0952 |
| ア | アルコールは特に弱く |  | 0.100 |
| 起き | 夜はトイレに行きたくて＊時間おきに起き | + | 0.105 |
| 味覚 | 味覚は＊割くらいしか戻ってきていない |  | 0.111 |
| 血圧 | オプスミットで結構＊＊台まで血圧落ち |  | 0.111 |
| お薬 | お薬とかではとくに副作用みたいなの |  | 0.118 |
| 転倒 | 神経内科の薬は、飲み始めたら転倒 | + | 0.125 |
| 便はきのう少しで | 便 | + | 0.125 |
| 小 | 小さいときは喘息 |  | 0.125 |
| 急に | 手術後、急に左眼の眼圧が上がり |  | 0.133 |
| 痛く | 手術終わって＊日くらいは痛く | + | 0.143 |
| あ | あまり眠れない |  | 0.143 |
| がん | 抗がん剤始まってから歯が浮く |  | 0.143 |
| 症状 | ぶつぶつが出るといった症状 | + | 0.154 |
| 後から | シスプラチン投与したすぐ後からお腹痛く |  | 0.158 |
| 便秘 | 便秘も＊週間くらいひどい | + | 0.167 |
| 少 | 少し食欲なく |  | 0.167 |
| 抗生剤 | ＊抗生剤について＊とくに気になる症状 |  | 0.167 |
| 気持ち悪くなっ | モーラステープは貼ったら気持ち悪く | + | 0.176 |
| 副作用 | お薬とかではとくに副作用みたいなの | + | 0.176 |
| 食事 | 食事もあんまりとれない | + | 0.182 |
| 出血 | 手術で出血が止まらなく | + | 0.182 |
| ダメ | 食べ物のにおいとかダメ |  | 0.182 |
| 腎臓 | 腎臓が片方機能してない |  | 0.182 |
| すっきり便 | 便 | + | 0.200 |
| 胃が痛くな | デカドロン飲んでたら、胃が痛く | + | 0.200 |
| ふわふわ | 薬＊マグセント＊使い始めてからふわふわ | + | 0.211 |
| お腹痛く | シスプラチン投与したすぐ後からお腹痛く | + | 0.211 |
| 時間おき | 夜はトイレに行きたくて＊時間おきに起き |  | 0.211 |
| しびれ | ベルケイド使ったときはしびれ | + | 0.214 |
| 眠気 | 薬飲み始めて、眠気 | + | 0.222 |
| 目覚め | 夜は＊＊＊時間おきに目覚め | + | 0.231 |
| 頭ぼーっとして | 造影剤使った後は頭ぼーっと | + | 0.231 |
| 吐き気 | ＊オキノームにより＊吐き気 | + | 0.231 |
| 下がっ | サチュレーションが下がって |  | 0.231 |
| 血圧は | 血圧は入院してから下がって |  | 0.231 |
| 貼ったら | モーラステープは貼ったら気持ち悪く |  | 0.235 |
| 熱が下が | 熱 | + | 0.250 |
| 出血 | 出血しやすい体質 | + | 0.250 |
| 震え | 手は昨日から震え | + | 0.250 |
| 喘息 | 小さいときは喘息 | + | 0.250 |
| 痛み | 痛みは動くと少し | + | 0.250 |
| 脱毛 | とても脱毛が進み | + | 0.250 |
| ステロイドが怖い | 怖い | + | 0.250 |
| 口内炎などの症状 | 症状 | + | 0.250 |
| 少し | 少し顔が赤い感じ |  | 0.250 |
| 夜 | 夜に痛く |  | 0.250 |
| 手は | 手は昨日から震え |  | 0.250 |
| 尿酸 | 尿酸も血圧も高い |  | 0.250 |
| 膵 | 膵臓がん |  | 0.250 |
| ひどい | 便秘も＊週間くらいひどい |  | 0.250 |
| シスプラチ | シスプラチン投与したすぐ後からお腹痛く |  | 0.263 |
| お痛じ | お痛じは昨日出ていなく | + | 0.273 |
| 吐き気 | 抗がん剤のあとに吐き気 | + | 0.273 |
| お通じ止 | ビオフェルミンはお通じ止まっ | + | 0.286 |
| 肺炎 | 肺炎の薬も発疹 | + | 0.286 |
| 発疹 | 肺炎の薬も発疹 | + | 0.286 |
| 赤く | 赤くブワーッと | + | 0.286 |
| 歯が浮く | 抗がん剤始まってから歯が浮く | + | 0.286 |
| 喘息 | 喘息は子供の時 | + | 0.286 |
| 少し | 少し気持ち悪く |  | 0.286 |
| 少し | 少し気持ち悪い |  | 0.286 |
| 調子わるく | 新しい薬始めるとだいたい調子わるく | + | 0.294 |
| 注意するような副作用 | 副作用 | + | 0.300 |
| 抗生剤で | 抗生剤でちょっと下痢っぽく |  | 0.308 |
| ざ瘡 | ざ瘡様皮膚炎 | + | 0.333 |
| 口内炎が痛く | 痛く | + | 0.333 |
| 食欲は | 食欲はやっぱりない | + | 0.333 |
| 咳した | 咳 | + | 0.333 |
| 腹水たまって | 腹水 | + | 0.333 |
| ＊の熱 | 熱 | + | 0.333 |
| 手を入れてげっぷ出 | げっぷ | + | 0.333 |
| すーごい眠くて | ＊＊＊＊あるとき＊すーごい眠く | + | 0.333 |
| 手を打っただけ | ちょっと手を打った | + | 0.333 |
| 気になる症状 | ＊抗生剤について＊とくに気になる症状 | + | 0.333 |
| 耳鳴りも下痢 | 下痢 | + | 0.333 |
| 気分悪さ | ＊ヘパリン＊特に気分悪さ | + | 0.333 |
| 便秘も、下痢 | 便秘 | + | 0.333 |
| 唾液 | 唾液が出ない |  | 0.333 |
| ヘパリン | ＊ヘパリン＊特に気分悪さ |  | 0.333 |
| め | めまい |  | 0.333 |
| 味が薄い | 味が薄いというかしない | + | 0.364 |
| お通じは便秘気味 | お通じ | + | 0.375 |
| 目覚め | 夜は何回か目覚め | + | 0.375 |
| 口内炎などの症状 | 口内炎 | + | 0.375 |
| 何回か | 夜は何回か目覚め |  | 0.375 |
| 夜なかなか | なかなか眠れない |  | 0.375 |
| 麻酔で | 麻酔で気持ち悪く |  | 0.375 |
| 乗り物酔い | 乗り物酔いとかの気持ち悪さ | + | 0.385 |
| 気持ち悪さ | 乗り物酔いとかの気持ち悪さ | + | 0.385 |
| ＊＊＊くらいし | ＊＊＊くらいしか息ができていない感じ |  | 0.389 |
| お通じが | お通じが産後出てなく | + | 0.400 |
| 痛い | のどは痛い | + | 0.400 |
| 風邪ひいた | 風邪 | + | 0.400 |
| 眠気が続い | 眠気 | + | 0.400 |
| 痛み | 痛みは少し | + | 0.400 |
| 悪く | 悪くなった |  | 0.400 |
| お腹はったり痛かった | 痛かった | + | 0.400 |
| 風邪引いた | 風邪 | + | 0.400 |
| おかしい | 眼の見え方がおかしい |  | 0.400 |
| ちょっと | ちょっと眠れなかった |  | 0.400 |
| 便が硬くて出なく | 胃薬飲むようになったら、便が硬くて出なく | + | 0.400 |
| トイレに行きたく | 夜はトイレに行きたくて＊時間おきに起き |  | 0.421 |
| 残尿感は減った | 残尿感 | + | 0.429 |
| 副作用 | ＊＊＊は副作用 | + | 0.429 |
| あまり | あまり眠れない |  | 0.429 |
| 麻酔で | 麻酔で呼吸困難 |  | 0.429 |
| 夜中に | 夜中に目が覚め |  | 0.429 |
| ちょっと | ちょっと手を打った |  | 0.444 |
| 化学療法のあとは | 化学療法のあとはお通じが出にくかった |  | 0.444 |
| ちょっと | ちょっと気持ち悪い |  | 0.444 |
| お腹がゆるくな | 抗生物質でお腹がゆるく | + | 0.455 |
| 抗生物質で | 抗生物質でお腹がゆるく |  | 0.455 |
| 食べられない | 半分くらいしか食べられない | + | 0.462 |
| 怖か | 怖かった | + | 0.500 |
| 名前忘れ | 忘れ | + | 0.500 |
| ンの痛み | 痛み | + | 0.500 |
| かゆみ | 輸液でかゆみ | + | 0.500 |
| 眠れない | なかなか眠れない | + | 0.500 |
| 痛く | 夜に痛く | + | 0.500 |
| 出血がこわい | こわい | + | 0.500 |
| 皮膚炎 | ざ瘡様皮膚炎 | + | 0.500 |
| 口内炎が痛く | 口内炎 | + | 0.500 |
| お通じは便秘気味 | 便秘気味 | + | 0.500 |
| 眠く | 昼間眠く | + | 0.500 |
| しびれ | しびれは少し | + | 0.500 |
| 熱が | 熱 | + | 0.500 |
| 咳と | 咳 | + | 0.500 |
| 熱が | 熱 | + | 0.500 |
| 痰が | 痰 | + | 0.500 |
| 耳鳴りも下痢 | 耳鳴り | + | 0.500 |
| 熱が | 熱 | + | 0.500 |
| 副作用 | 副作用強そう | + | 0.500 |
| 皮疹出た | 皮疹 | + | 0.500 |
| 痛み | 痛み強い | + | 0.500 |
| お腹はったり痛かった | お腹はった | + | 0.500 |
| 輸液で | 輸液でかゆみ |  | 0.500 |
| 緊 | 緊張 |  | 0.500 |
| 昼間 | 昼間眠く |  | 0.500 |
| リウ | リウマチ |  | 0.500 |
| 吐き | 吐き出す |  | 0.500 |
| 睡 | 睡眠 |  | 0.500 |
| お腹はゆるかった | 手術したときもお腹はゆるかった | + | 0.533 |
| ＊＊＊時間おき | 夜は＊＊＊時間おきに目覚め |  | 0.538 |
| お通じが出にくかった | 化学療法のあとはお通じが出にくかった | + | 0.556 |
| 気持ち悪い | ちょっと気持ち悪い | + | 0.556 |
| 息ができていない感じ | ＊＊＊くらいしか息ができていない感じ | + | 0.556 |
| 眠れない | あまり眠れない | + | 0.571 |
| 眠れない | あまり眠れない | + | 0.571 |
| 呼吸困難 | 麻酔で呼吸困難 | + | 0.571 |
| 目が覚め | 夜中に目が覚め | + | 0.571 |
| リウマチの痛み | リウマチ | + | 0.571 |
| ところは少し赤くな | 注射したところは少し赤く | + | 0.583 |
| 脊髄からくるよ | 脊髄からくるような吐き気 |  | 0.583 |
| ＊夜中に起 | 夜中に起き | + | 0.600 |
| 食べられない | あんまり食べられない | + | 0.600 |
| しびれ | しびれは足 | + | 0.600 |
| 覚えられない | なかなか覚えられない | + | 0.600 |
| 眠れなかった | ちょっと眠れなかった | + | 0.600 |
| 眠れて | 眠れてない | + | 0.600 |
| 左眼の眼圧が上がり | 手術後、急に左眼の眼圧が上がり | + | 0.600 |
| 産後出てなく | お通じが産後出てなく |  | 0.600 |
| のどは | のどは痛い |  | 0.600 |
| 眼の見え方が | 眼の見え方がおかしい |  | 0.600 |
| 血圧も高い | 尿酸も血圧も高い | + | 0.625 |
| 気持ち悪く | 麻酔で気持ち悪く | + | 0.625 |
| 腎機能悪く | 腎機能悪くなった | + | 0.625 |
| 動くと少し | 痛みは動くと少し |  | 0.625 |
| あたまの骨 | あたまの骨がない |  | 0.625 |
| ウムが低い | カルシウムが低い |  | 0.625 |
| おぼえられない | なかなかおぼえられない | + | 0.636 |
| 食べ物のにおい | 食べ物のにおいとかダメ |  | 0.636 |
| おしっこの色が | 若干おしっこの色が濃い |  | 0.636 |
| 糖尿 | 糖尿病 | + | 0.667 |
| 糖尿 | 糖尿病 | + | 0.667 |
| おつうじ安定 | おつうじ安定しない | + | 0.667 |
| 色が黒くなっ | 色が黒く | + | 0.667 |
| 食事は | 食事 | + | 0.667 |
| 染み | 染みて | + | 0.667 |
| 肝機能が悪くなっ | 肝機能が悪くなっちゃった | + | 0.667 |
| 寝にくか | 寝にくかった | + | 0.667 |
| 食欲なく | 少し食欲なく | + | 0.667 |
| 乾燥し | 乾燥 | + | 0.667 |
| 痺れはひどく | 痺れはひどくなって | + | 0.667 |
| お腹が張ってしまい | お腹が張って | + | 0.667 |
| 出血す | 出血 | + | 0.667 |
| 感染 | 感染症 | + | 0.667 |
| ＊痒み | 痒み | + | 0.667 |
| ちょっと下痢っぽく | 抗生剤でちょっと下痢っぽく | + | 0.692 |
| シリンで副作用 | ワイドシリンで副作用 | + | 0.700 |
| 寝られなか | 寝られなかった | + | 0.714 |
| 気持ち悪くなっ | 気持ち悪く | + | 0.714 |
| 便がでなくなっ | 便がでなく | + | 0.714 |
| 気持ち悪く | 少し気持ち悪く | + | 0.714 |
| お腹が圧迫され | お腹が圧迫 | + | 0.714 |
| 気持ち悪い | 少し気持ち悪い | + | 0.714 |
| 痛みは強く | 痛みは強くなり | + | 0.714 |
| ベルケイド使ったとき | ベルケイド使ったときはしびれ |  | 0.714 |
| ＊時間おきくらい | ＊時間おきくらいに起き |  | 0.727 |
| 痛かっ | 痛かった | + | 0.750 |
| ふるえた | ふるえ | + | 0.750 |
| 足がびりびりして | 足がびりびり | + | 0.750 |
| 顔が赤い感じ | 少し顔が赤い感じ | + | 0.750 |
| お通じは | お通じ | + | 0.750 |
| しびれや | しびれ | + | 0.750 |
| 顔も丸くむく | 顔も丸くむくんだ |  | 0.750 |
| リズムが乱れ | 生活リズムが乱れ |  | 0.750 |
| 食事とれてない | 全然食事とれてない | + | 0.778 |
| 血圧が落 | 血圧が落ち | + | 0.800 |
| あおたんが | あおたん | + | 0.800 |
| 咽頭がん | 中咽頭がん | + | 0.800 |
| 胃液を吐い | 胃液を吐いて | + | 0.833 |
| 血糖下がっち | 血糖下がっ | + | 0.833 |
| 便が出てな | 便が出てない | + | 0.833 |
| おトイレの回数が多か | おトイレの回数が多かった | + | 0.833 |
| 腰がいたくて | 腰がいたく | + | 0.833 |
| 咳がひどくて | 咳がひどく | + | 0.833 |
| 血圧が低くな | 血圧が低く | + | 0.833 |
| 喉がいたくて | 喉がいたく | + | 0.833 |
| 声がかすれて | 声がかすれ | + | 0.833 |
| アレルギー＊ | アレルギー | + | 0.833 |
| 気持ち悪い様 | 気持ち悪い | + | 0.833 |
| 体調は変わ | 体調は変わり | + | 0.833 |
| 少しよろめい | 少しよろめいて | + | 0.857 |
| 頭がぼんやりし | 頭がぼんやり | + | 0.857 |
| ききもちわるく | きもちわるく | + | 0.857 |
| 便がたまってい | 便がたまって | + | 0.857 |
| ちょっと腫れて | ちょっと腫れ | + | 0.857 |
| 覚えていませ | 覚えていません | + | 0.857 |
| 白血球も下がっ | 白血球も下がって | + | 0.875 |
| 全身がかさかさで | 全身がかさかさ | + | 0.875 |
| ほてりはちょっとあ | ほてりはちょっと | + | 0.889 |
| 目覚めが悪い感じ | 朝目覚めが悪い感じ | + | 0.889 |
| 足の裏がずっと痺れて | 足の裏がずっと痺れ | + | 0.900 |
| おなかはぐるぐる動い | おなかはぐるぐる動いて | + | 0.909 |
| 傷口はむずむず痛い感 | 傷口はむずむず痛い感じ | + | 0.909 |

The results of partial matches for four folds during cross-validation of 1200 trainings were examined to determine if symptom information was missing or not. Extracted terms with “+” for “Symptom (+)” are those for which symptom information remained, even if only partially. The results show that the number of missing symptoms increases when the similarity is less than 0.667. The asterisks in terms extracted by the system or researchers represent infrequent characters which were converted to unknown keys during the process of the information processing in BERT.
