# Supplementary material for: Performance Improvement of a Natural Language Processing Tool for Extracting Patient Narratives Related to Medical States From Japanese Pharmaceutical Care Records by Increasing the Amount of Training Data: Natural Language Processing Analysis and Validation Study
Source: JMIR Med Inform. 2025 Mar 4;13:e68863. doi: 10.2196/68863 (PMC11920660; doi:10.2196/68863)
Supplement: Multimedia Appendix 2 [file medinform_v13i1e68863_app2.docx]

**Multimedia Appendix 2.** Number of errors and the ratio of errors to total extraction in the subcategories of error 3.

| **Subgroup** | Number of errors in 1200-record training (%) | Number of errors in 3600-record training (%) | Number of errors in 12,004-record training (%) |
| --- | --- | --- | --- |
| Total errors of cause category 3 | 209 | 118 | 114 |
| Error 3a: Determined from other information | 105 (3.5) | 74 (3.1) | 60 (2.5) |
| Error 3b: Determined from positive expressions | 60 (2.0) | 15 (0.6) | 24 (1.0) |
| Error 3c: Determined from negative expressions | 29 (1.0) | 13 (0.5) | 16 (0.7) |
| Error 3d: Researcher error | 10 (0.3) | 11 (0.5) | 11 (0.4) |
| Error 3e: Coexistence of positive and negative expressions | 5 (0.2) | 5 (0.2) | 3 (0.1) |

Total extraction = 2963 for 1200-record training, 2387 for 3600-record training and 2446 for 12,004-record training

P-N: Positive-negative

With the increase in the training amount from 1200 to 12,004 records, significant improvement was seen in this order: “determined from other information (error 3a)”, “determined from positive expressions (error 3b)”, “determined from negative expressions (error 3c)”.

Error 3a included those that did not directly describe the presence or absence of symptoms but could be inferred, as well as those that were difficult to determine, which could be judged differently by researchers. The former example is “diabetes” included in “I take my diabetes medicine correctly every day,” and the latter example is “Dizziness” included in “Dizziness or something like that.” Error 3b and error 3c are errors that occur when the patient directly refers to the presence or absence of an expression, such as “The stomach discomfort has resolved” or “The hiccups have stopped.” Increasing the training amount improved errors 3a, 3b, and 3c, while error 3d increased from 0.3% (10/2963) to 0.4% (11/2446).
