# Supplementary material for: Performance Improvement of a Natural Language Processing Tool for Extracting Patient Narratives Related to Medical States From Japanese Pharmaceutical Care Records by Increasing the Amount of Training Data: Natural Language Processing Analysis and Validation Study
Source: JMIR Med Inform. 2025 Mar 4;13:e68863. doi: 10.2196/68863 (PMC11920660; doi:10.2196/68863)
Supplement: Multimedia Appendix 3 [file medinform_v13i1e68863_app3.docx]

**Multimedia Appendix 3.** Number of errors and the ratio of errors to total extraction in the subcategories of error 3b and 3c focused on the position of positive-negative expressions.

| **Subgroup** | Number of errors in 1200-record training (%) | Number of errors in 3600-record training (%) | Number of errors in 12,004-record training (%) |
| --- | --- | --- | --- |
| Total errors of cause category 3b and 3c | 89 | 28 | 40 |
| Error 3bc-1-1: Immediately before or/and after the symptom | 59 (2.0) | 20 (0.8) | 21 (0.9) |
| Error 3bc-1-2: Included within the extracted terms | 14 (0.5) | 3 (0.1) | 7 (0.3) |
| Error 3bc-1-3: One or two words ^a^ are in between from the extracted terms | 9 (0.3) | 4 (0.2) | 6 (0.3) |
| Error 3bc-1-4: More than three words ^a^ are in between from the extracted terms | 4 (0.1) | 1 (0.0) | 4 (0.2) |
| Error 3bc-1-5: Determination with reference to the P-N ^b^ classification of another extracted term in the same sentence | 3 (0.1) | 0 (0.0) | 2 (0.1) |

Total extraction = 2963 for 1200-record training, 2387 for 3600-record training and 2446 for 12,004-record training

^a^Postpositional particles, auxiliary verbs, auxiliary adjectives, and degree expressions modifying symptoms were excluded from the count.

^b^P-N: Positive-Negative
