# Supplementary material for: Performance Improvement of a Natural Language Processing Tool for Extracting Patient Narratives Related to Medical States From Japanese Pharmaceutical Care Records by Increasing the Amount of Training Data: Natural Language Processing Analysis and Validation Study
Source: JMIR Med Inform. 2025 Mar 4;13:e68863. doi: 10.2196/68863 (PMC11920660; doi:10.2196/68863)
Supplement: Multimedia Appendix 4 [file medinform_v13i1e68863_app4.docx]

**Multimedia Appendix 4.** Number of errors and the ratio of errors to total extraction in the subcategories of error 3b and 3c focused on the way of positive-negative expressions.

| **Subgroup** | Number of errors in 1200-record training (%) | Number of errors in 3600-record training (%) | Number of errors in 12,004-record training (%) |
| --- | --- | --- | --- |
| Total errors of cause category 3b and 3c | 89 | 28 | 40 |
| Error 3bc-2-1: Simple positive or negative expressions | 57 (1.9) | 16 (0.7) | 18 (0.7) |
| Error 3bc-2-2: Included within the extracted terms | 11 (0.4) | 3 (0.1) | 6 (0.3) |
| Error 3bc-2-3: Mild negative expressions | 11 (0.4) | 3 (0.1) | 5 (0.2) |
| Error 3bc-2-4: Negated positive or negative expressions | 5 (0.2) | 6 (0.3) | 8 (0.3) |
| Error 3bc-2-5: Determination with reference to the P-N ^a^ classification of another extracted term in the same sentence | 3 (0.1) | 0 (0.0) | 2 (0.1) |
| Error 3bc-2-6: Reversal of P-N ^a^ classification by partial match | 2 (0.1) | 0 (0.0) | 1 (0.0) |

Total extraction = 2963 for 1200-record training, 2387 for 3600-record training and 2446 for 12,004-record training

^a^P-N: Positive-Negative

The subcategory which shows no improvement after 3600-record training and had the largest error rate at 12,004-record training was for “simple positive or negative expressions (error 3bc-2-1)”. Since each validation data contained many expressions which only appeared in each training amount, it was difficult to perform further detailed classification. Common expressions which appeared both in the validation data at 3600 and 12,004-record training were “I sometimes ~” (“~koto ga aru” in Japanese) and “no problem” (“daijoubu” in Japanese).
